# Differentiating Non-Motor Symptoms in Parkinson's Disease from Controls and Hemifacial Spasm

**DOI:** 10.1371/journal.pone.0049596

**Published:** 2013-02-11

**Authors:** Ming-Hui Yong, John C. Allen, Kumar M. Prakash, Eng-King Tan

**Affiliations:** 1 Department of Neurology, National Neuroscience Institute, Singapore, Singapore; 2 Duke-NUS Graduate Medical School, Singapore, Singapore; Oslo University Hospital, Norway

## Abstract

**Background and Aims:**

Non-motor symptoms (NMS) are important manifestations of Parkinson's disease (PD) that reduce patients' health-related quality of life. Some NMS may also be caused by age-related changes, or manifested as a psychological reaction to a chronic neurological condition. This case-control study compared the NMS burden among PD patients, healthy controls and hemifacial spasm (HFS) patients. In addition, we determined the NMS that discriminated between PD and non-PD subjects.

**Methods:**

425 subjects were recruited from a tertiary hospital in Singapore (200 PD patients, 150 healthy controls and 75 HFS patients). NMS burden in subjects was measured using the Non-Motor Symptoms Scale (NMSS).

**Results:**

NMSS total score was significantly higher in PD patients (37.9±2.6) compared to healthy controls (11.2±0.9) (*p*<0.0001) and HFS patients (18.0±2.1) (*p*<0.0001). In addition, NMSS total score was significantly higher in HFS patients compared to healthy controls (*p* = 0.003). PD patients experienced a higher NMS burden than healthy controls in all domains, and a higher NMS burden than HFS patients in all but attention/memory and urinary domains. NMS burden for HFS and healthy controls differed only in the sleep/fatigue and urinary domains. Using stepwise logistic regression, problems of ‘constipation’, ‘restless legs’, ‘dribbling saliva’, ‘altered interest in sex’ and ‘change in taste or smell’ were found to have significant discriminative power in differentiating between PD patients and healthy controls and between PD patients and HFS patients.

**Conclusion:**

PD patients experienced a greater overall NMS burden compared to both healthy controls and HFS patients. HFS patients demonstrated a higher NMS burden than controls, and some NMS may be common to chronic neurological conditions while others are more specific to PD. Differentiating patients using NMS domains may help refine the clinical management of NMS in PD patients.

## Introduction

Non-motor symptoms (NMS) are important manifestations of Parkinson’s disease (PD) in addition to the cardinal motor symptoms of bradykinesia, tremor, rigidity and postural instability, and some eventually become prominent causes of disability [1], [2]. The current understanding is that NMS results from Lewy body pathology involving regions of the nervous system outside the substantia nigra, [3] which begins in the olfactory bulb and medulla, and progresses through the central nervous system from the medulla in a predictable ascending sequence described by the Braak stages [3]–[5]. The peripheral autonomic nervous system is also involved [3], [6]. Sufficiently disrupted neurotransmitter pathways would theoretically result in the clinical manifestation of NMS. Antiparkinsonian medications may also precipitate, exacerbate or relieve NMS, [7], [8] adding complexity to NMS characterization and treatment. NMS have been shown to have a significant impact on PD patients' health-related quality of life that may be greater than that of motor symptoms [9]–[13].

NMS characterization is not straightforward: The range of NMS is vast, and clinical correlation with neuropathological findings is partial and not fully established [5]. Furthermore, NMS may be caused by normal age-related changes or other diseases, or develop as a psychological reaction to a chronic disease. The Non-Motor Symptoms Scale (NMSS) is a clinically validated instrument that comprehensively assesses the burden of 30 NMS relevant in PD [14], [15]. It calculates a score for each NMS as a function of severity and frequency to reflect its burden. It is suitable for assessing a wide range of NMS and evaluating which ones clinically manifest differentially in PD patients. To date, there exist few case-controls studies using the NMSS to compare the NMS burden between PD patients and healthy controls, [16], [17] and none have investigated its utility in detecting NMS which may develop as a reaction to suffering from a chronic neurological disease.

We utilized the NMSS to compare the NMS burden between non-demented idiopathic PD patients, healthy controls and hemifacial (HFS) patients, and to identify which of the 30 NMS evaluated in the scale best discriminate between PD patients and the two groups of non-PD subjects. HFS was chosen as a comparator because it is a chronic, localized neurologic disease which is not known to have pathology such as that exhibited in a generalized neurodegenerative disorder like PD that could directly cause NMS.

## Methods

### Subjects

200 idiopathic Parkinson's disease (PD) patients, diagnosed according to the UK PD Brain Bank criteria, [18] were recruited from the movement disorder outpatient clinics in Singapore General Hospital (SGH), a tertiary referral center. PD patients with significant cognitive impairment, defined as scoring 5 points or less (out of a maximum of 10 points) on our locally validated Elderly Cognitive Assessment Questionnaire (ECAQ), [19] were excluded. 150 healthy controls frequency-matched for age (± 5 years) and sex also participated. A third group of 75 similarly matched patients diagnosed with hemifacial spasm (HFS) (based on the presence of tonic and/or clonic contractions of facial muscles innervated by the ipsilateral facial nerve) [20] and receiving botulinum toxin treatment from the SGH movement disorders outpatient clinic was also recruited. Only subjects of Asian ethnicity were included. Any subject with a severe chronic debilitating condition (e.g. renal failure requiring dialysis, congestive cardiac failure, diabetes mellitus with advanced complications, other CNS disorders), or with terminal illness was excluded. Hypertension, hyperlipidemia, controlled diabetes mellitus and ischemic heart disease are common conditions that do not cause excessive distress in general, and these conditions were also not excluded. The study was approved by the SingHealth Centralised Institutional Review Board.

### Data collection and assessments

Demographic data was collected from subjects. Non-motor symptoms (NMS) were assessed using the Non-Motor Symptoms Scale (NMSS) [14]. This scale evaluates 30 NMS relevant in PD, grouped into 9 domains (cardiovascular domain, sleep/fatigue, mood/cognition, perceptual problems/hallucinations, attention/memory, gastrointestinal, urinary, sexual function and miscellaneous). Each item in the NMSS rates a NMS according to its severity (scored from 0 to 3) and frequency (scored from 1 to 4) over the past month, and these two ratings are multiplied to give a final item score representing the burden of that symptom. A domain score is calculated for each domain as the sum of the item scores within that domain. The NMSS total score, the sum of all the item scores, reflects the overall NMS burden in a subject. The presence of each NMS in subjects was also coded.

PD patients underwent further clinical assessment for disease severity with Part III (motor section) of the Unified Parkinson's Disease Rating Scale (mUPDRS) [21] and the modified Hoehn and Yahr (H&Y) staging scale [22]. The levodopa equivalent daily dose (LEDD) was calculated according to standardized formulae, using terms for levodopa, dopamine agonists and entacapone: LEDD (mg/day)  =  ([ L-dopa (mg)] + [Controlled release L-dopa (mg)] ×0.75) (×1.33 for L-dopa doses taken together with entacapone) + [pramipexole (mg) ×100] + [ropinirole (mg) ×20] + [piribedil (mg) ×1] + [bromocriptine (mg) ×10] [23].

### Statistical analysis

Baseline characteristics for continuous variables were compared across all groups using the Kruskal-Wallis test, and pair-wise comparisons were performed using the Wilcoxon rank-sum test. Categorical variables were compared using the chi-square test. Continuous variables for baseline characteristics are summarized as mean ± standard deviation; categorical variables are summarized as percentages.

Frequency distribution plots of NMSS scores, whether for each item, domain or total score, consistently exhibited highly right-skewed, heavy tailed distributions resembling those of over-dispersed counting distributions. In the search for an acceptable statistical model, various error distributions and link functions were evaluated in the context of a generalized linear model (GLIM). The best fitting (lowest AIC) model for comparing NMSS scores (item, domain and total) between the three groups incorporated the negative binomial error distribution in conjunction with the log link function. An analysis comparing NMSS total score among the three groups incorporated age, sex, presence of hypertension, hyperlipidemia, diabetes mellitus and ischemic heart disease in the model as potential confounders. The only one found to be statistically significant was age. Because an attempt was made to match groups on age and sex, both variables were included in the final model. The Tukey-Kramer approach was used to adjust the type I error for multiple pair-wise comparisons, and tabulated p-values reflect the adjustment. Comparison of NMSS scores between PD patients of the different disease (H&Y) stages was performed using the same methodology. Logistic regression analysis was used to compare prevalence of NMS among groups, controlling for age and sex. In addition to age and sex, diabetes mellitus was a statistically significant factor in the comparison of NMSS scores and prevalence of the symptom of ‘problems having sex’ in the sexual function domain. All reported prevalence values and mean NMSS scores are model-based, predicted proportions and least squares means (± standard errors), respectively, adjusted for covariates. Stepwise logistic regression (significance level to enter  =  significance level to remove  = 0.05) was used to select non-motor symptoms in the NMSS which best discriminated between PD patients and healthy controls, and between PD patients and HFS patients.

In a subgroup analysis of PD patients, Spearman's rank correlation (*rho*) was used to investigate univariate associations between the NMSS total score and selected variables. Analysis of covariance was used to determine clinical factors associated with NMSS total scores and domain scores–H&Y stage was grouped into 3 classes: stages 1–1.5, 2–2.5, and 3–5.

Statistical significance was set at *p*≤0.05. All statistical analyses were performed using the SAS software (version 9.2; North Carolina: SAS Institute Inc.).

## Results

### Demographic data and PD patient characteristics

The mean age was 64.4±9.7 years in the PD patient group, 60.7±11.3 in the healthy control group, and 62.2±11.1 in the HFS patient group. 54.5% of PD patients, 52.0% of healthy controls and 49.3% of HFS patients were male. Demographic and comorbidity data are summarized in Table 1.

**Table 1:** Demographic data.

Demographic data	Parkinson's disease patients	Healthy controls	Hemifacial spasm patients	p-value
	(n = 200)	(n = 150)	(n = 75)	(K-W or chi-square)
Age (mean ± SD) a [min, max]	64.4±9.7 [39, 84]	60.7±11.3 [35, 87]	62.2±11.1 [31, 85]	**0.004**
Sex (% male)	54.5	52.0	49.3	0.730
Hypertension (%) a, b	41.5	29.3	46.7	**0.017**
Hyperlipidemia (%)	33.5	24.7	33.3	0.170
Diabetes mellitus (%) a, b	18.0	7.3	16.0	**0.014**
Ischemic heart disease (%)	7.5	6.0	4.0	0.557

a Significant difference between healthy control group and PD patient group (p<0.05).

b Significant difference between control group and disease control group (p<0.05).

In the PD patient group, mean disease duration was 6.0±4.5 years and mean mUPDRS score was 28.4±13.2. The median H&Y stage was 2.5. 16.5% of PD patients were in H&Y stages 1 – 1.5, 60.5% in stages 2 – 2.5, 21% in stage 3, and 2% in stages 4 – 5. 78.5% of patients were on levodopa and 20.5% were on a dopamine agonist; 11.5% were on both levodopa and a dopamine agonist. 11.0% were on neither levodopa nor a dopamine agonist. The mean levodopa equivalent daily dose (LEDD) was 369.2±295.5 mg/day. For patients on dopamine agonists, the mean dopamine agonist daily dose (in levodopa-equivalent units) was 67.8±48.5 mg/day. The mean disease duration in the HFS patient group was 10.9±5.3 years.

### NMS burden

Least squares (LS) mean NMSS total and domain scores adjusted for age and sex are presented in Table 2. The adjusted mean NMSS total score (reflecting overall NMS burden) was significantly higher in the PD patients compared to healthy controls (37.9±2.6 vs. 11.2±0.9 respectively, *p*<0.0001) and HFS patients (37.9±2.6 vs. 18.0±2.1, *p*<0.0001). The adjusted mean NMSS total score in HFS patients was also significantly higher than that of healthy controls (*p* = 0.003).

**Table 2:** Comparisons of estimated NMSS total and domain scores^1^ by study group.

	Study Group			F-test *p*-value	Pair-wise Comparison *p*-values^3^			
	PD	C	HFS		PD vs C	PD vs HFS		HFS vs C
NMSS total score	37.9 (±2.6)	11.2 (±0.9)	18.0 (±2.1)	**<0.0001**	**<0.0001**	**<0.0001**		**0.003**
Domains								
Cardiovascular domain	1.0 (±0.2)	0.3 (±0.1)	0.3 (±0.1)	**<0.0001**	**<0.0001**	**0.001**	0.884	
Sleep/fatigue domain	8.2 (±0.8)	2.5 (±0.3)	4.3 (±0.7)	**<0.0001**	**<0.0001**	**0.002**	**0.016**	
Mood domain	9.2 (±1.2)	1.9 (±0.3)	3.5 (±0.8)	**<0.0001**	**<0.0001**	**0.001**	0.064	
Perceptual problems/hallucinations domain	1.0 (±0.2)	0.05 (±0.02)	0.1 (±0.1)	**<0.0001**	**<0.0001**	**0.0003**	0.642	
Attention/memoryDomain	3.5 (±0.3)	1.9 (±0.2)	2.9 (±0.5)	**0.0003**	**0.0002**	0.536	0.091	
Gastrointestinal domain	3.2 (±0.5)	0.2 (±0.1)	0.3 (±0.1)	**<0.0001**	**<0.0001**	**<0.0001**	0.924	
Urinary domain	5.1 (±0.6)	1.8 (±0.3)	3.4 (±0.6)	**<0.0001**	**<0.0001**	0.158	**0.016**	
Sexual function domain^2^	1.4 (±0.2)	0.8 (±0.1)	0.7 (±0.2)	**0.003**	**0.010**	**0.028**	0.953	
Miscellaneous domain	5.1 (±0.6)	1.6 (±0.2)	2.0 (±0.4)	**<0.0001**	**<0.0001**	**<0.0001**	0.648	

PD: Parkinson's disease patients; C: Healthy controls; HFS: Hemifacial spasm patients.

1 Estimated mean scores adjusted for age and sex (± standard error) using GLIM negative binomial model^ 2^Estimated percentages adjusted for age, sex and presence of diabetes mellitus (± standard error) using logistic regression model.

3 Tukey-Kramer adjusted p-values.

All of the domain scores were significantly higher in PD patients than in healthy controls. Sleep/fatigue and urinary domain scores were significantly higher in the HFS group compared to healthy controls. Item scores were higher in HFS patients than in healthy controls for the symptoms of ‘fatigue’ (1.8±0.3 vs. 0.8±0.1, *p* = 0.003), ‘forget to do things’ (1.1±0.2 vs 0.6±0.1, *p* = 0.050) and ‘urinary urgency’ (1.1±0.3 vs. 0.4±0.1, *p* = 0.023). Domain scores were significantly higher in the PD patient group compared to HFS patients, except for the attention/memory and urinary domains. The item scores for ‘fainting (secondary to orthostatic hypotension)’, ‘flat mood’, ‘delusions’, and ‘problems having sex’ did not differ significantly between the three groups. Symptoms with item scores that were significantly higher in PD patients compared to healthy controls (C) but not when compared to HFS patients included ‘difficulty falling or staying asleep’ (PD: 2.3±0.4, C: 0.9±0.2, HFS: 1.7±0.4; *p* = 0.001 for PD vs. C), ‘feel nervous’ (PD: 1.4±0.3, C: 0.3±0.1, HFS: 0.7±0.2; *p*<0.0001 for PD vs. C), ‘forgetting things or events’ (PD: 1.3±0.1, C: 0.8±0.1, HFS: 1.1±0.2; *p* = 0.005 for PD vs. C), ‘forgetting to do things’ (PD: 1.0±0.1, C: 0.6±0.1, HFS: 1.1±0.2; *p* = 0.020 for PD vs. C), ‘(urinary) urgency’ (PD: 1.3±0.2, C: 0.4±0.1, HFS: 1.1±0.3; *p* = 0.0002 for PD vs. C) and ‘urinary frequency’(PD: 1.3±0.2, C: 0.4±0.1, HFS: 1.0±0.3; *p* = 0.001 for PD vs. C). The symptoms of ‘fatigue’, ‘difficulty falling or staying asleep’, ‘nocturia’ and ‘pain’ contributed most to NMS burden for all three groups of subjects.

### NMS prevalence

The prevalence of NMSS domains (presence of at least one symptom in the respective domain) in our sample are shown in Table 3. Prevalence of cardiovascular symptoms, ‘delusions’, ‘concentration problems’, ‘forgetting things or events’, and of ‘pain’ did not differ between the three groups (data not shown). Sleep/fatigue symptoms (‘fatigue’ (46.7% vs. 28.9%, *p* = 0.009)) and ‘difficulty falling/staying asleep’ (46.8% vs. 23.1%, *p* = 0.001)), mood symptoms (‘loss of interest in the surroundings’ (18.4% vs. 6.0%, *p* = 0.006), ‘lack motivation’ (22.7% vs. 8.7%, *p* = 0.005), ‘feel nervous’ (25.9% vs. 9.7%, *p* = 0.002) and ‘difficulty experiencing pleasure’ (21.1% vs. 6.5%, *p* = 0.002)) and the symptom of ‘forget to do things’ (51.0% vs. 33.9%, *p* = 0.016) were more prevalent in HFS patients compared to controls.

**Table 3:** Comparisons of estimated prevalence^1^ (%) by NMSS domain.

	Study Group			F-test *p*-value	Pair-wise Comparison *p*-values^3^			
	PD	C	HFS		PD vs C	PD vs HFS		HFS vs C
Cardiovascular domain	27.1 (±3.2)	19.1 (±3.3)	19.6 (±4.6)	0.169	0.088	0.208	0.923	
Sleep/fatigue domain	83.9 (±2.6)	50.4 (±4.1)	68.1 (±5.4)	**<0.0001**	**<0.0001**	**0.005**	**0.013**	
Mood domain	68.6 (±3.3)	33.8 (±3.9)	58.8 (±5.7)	**<0.0001**	**<0.0001**	0.134	**0.0005**	
Perceptual problems/hallucinations domain	17.3 (±2.8)	3.2 (±1.4)	9.0 (±3.3)	**0.0005**	**0.0002**	0.092	0.071	
Attention/memoryDomain	69.6 (±3.3)	56.1 (±4.1)	64.7 (±5.6)	**0.042**	**0.012**	0.440	0.225	
Gastrointestinal domain	51.1 (±3.6)	10.1 (±2.5)	11.6 (±3.7)	**<0.0001**	**<0.0001**	**<0.0001**	0.722	
Urinary domain	67.5 (±3.5)	42.8 (±4.4)	53.3 (±6.1)	**0.0001**	**<0.0001**	**0.042**	0.166	
Sexual function domain^2^	55.1 (±4.5)	32.0 (±4.9)	32.1 (±6.1)	**<0.0001**	**<0.0001**	**0.002**	0.985	
Miscellaneous domain	67.5 (±3.4)	42.0 (±4.1)	42.5 (±5.8)	**<0.0001**	**<0.0001**	**0.0003**	0.945	

PD: Parkinson’s disease patients; C: Healthy controls; HFS: Hemifacial spasm patients.

1 Estimated percentages adjusted for age and sex (± standard error) using logistic model.

2 Estimated percentages adjusted for age, sex and presence of diabetes mellitus (± standard error) using logistic regression model.

3 Tukey-Kramer adjusted p-values.

### Clinical factors associated with NMS burden in PD patients

The NMSS total score (reflecting total NMS burden) correlated significantly with disease duration (*rho*  = 0.39, *p*<0.0001) and LEDD (*rho*  = 0.41, *p*<0.001). In a multivariate analysis of covariance investigating age, gender, disease duration, H&Y stage and LEDD, NMSS total score was positively associated with LEDD (*p* = 0.016) and H&Y stage (*p* = 0.0012). In the domain analysis, LEDD was positively associated with the sleep/fatigue (*p* = 0.017) and mood (*p* = 0.011) domain scores.

Stratification of NMSS scores based on H&Y stage is shown in Table 4. Sleep/fatigue, mood, gastrointestinal, sexual function and miscellaneous domain scores were demonstrated to increase between certain H&Y stages with statistical significance. However, it should be noted that, as described earlier, majority of the PD patients in our study were in H&Y stages 2–3 of the disease, and PD patients in H&Y stages 4–5 are underrepresented in our study.

**Table 4:** Comparisons of PD patient group estimated NMSS scores^1^ by H&Y stage.

	H&Y Stage Group			F-test *p*-value	Pair-wise Comparison *p*-values^2^					
	A	B	C		A vs B			B vs C		A vs C
	1–1.5	2−2.5	3−5							
	(n = 33)	(n = 121)	(n = 46)							
NMSS Total	19.7 (±3.0)	37.7 (±2.8)	57.0 (±6.9)	**<0.0001**	**0.0003**			**0.004**		**<0.0001**
Domains										
Cardiovascular domain	0.5 (±0.3)	0.9 (±0.2)	2.0 (±2.0)	0.071		0.347	0.051		**0.040**	
Sleep/fatigue domain	3.8 (±0.7)	7.8 (±0.7)	12.8 (±2.0)	**<0.0001**		**0.002**	**0.008**		**<0.0001**	
Mood domain	2.9 (±0.9)	9.3 (±1.4)	14.5 (±3.6)	**0.001**		**0.004**	0.126		**<0.0001**	
Perceptual problems /Hallucinations domain	1.0 (±0.7)	0.7 (±0.2)	1.8 (±1.0)	0.340		0.934	0.192		0.449	
Attention/memory domain	2.7 (±0.6)	3.5 (±0.4)	4.7 (±0.9)	0.204		0.345	0.536		0.091	
Gastrointestinal domain	2.0 (±0.7)	2.9 (±0.5)	5.5 (±1.4)	**0.041**		0.374	**0.031**		**0.003**	
Urinary domain	4.0 (±0.6)	5.4 (±0.6)	7.2 (±1.4)	0.162		0.254	0.166		0.106	
Sexual function domain	0.7 (±0.2)	1.4 (±0.2)	1.2 (±0.2)	**0.040**		**0.016**	0.426		0.062	
Miscellaneous domain	2.0 (±0.5)	5.5 (±0.7)	6.0 (±1.2)	**0.002**		**0.003**	0.722		**0.0004**	

1 Estimated mean scores adjusted for age and sex (± standard error) using GLIM negative binomial model.

2 Tukey-Kramer adjusted p-values.

### NMS that discriminated between PD patients and non-PD subjects

Both the presence and NMSS score of each NMS were included in the discriminative model, as a symptom due to PD may manifest in PD patients but not progress sufficiently for its burden to reach a threshold that discriminates between PD and non-PD subjects. The NMS whose presence or NMSS score possessed statistically significant discriminating power to differentiate between PD patients and healthy controls are presented in Table 5. For the model discriminating between PD patients and healthy controls, the three symptoms providing the greatest discriminative power were ‘fatigue’, ‘constipation’ and ‘dribbling saliva’.

**Table 5:** Selection of NMSS items by stepwise logistic regression^1^ to discriminate between PD patients (n = 200) and healthy controls (n = 150).

Selected NMSS items in order selected	Chi-Square Score	*p*-value	Cumulative area under the ROC curve	Odds Ratio	95% Wald Confidence Intervals	Wald *p*-value
NMSS score for ‘fatigue’	46.9	<0.0001	0.697	1.34	[1.17, 1.54]	< 0.0001
Presence of ‘constipation’	28.0	<0.0001	0.756	4.17	[1.83, 9.52]	0.001
Presence of ‘dribbling saliva’	20.6	<0.0001	0.791	23.81	[4.88, 111]	< 0.001
Presence of ‘altered sense of taste or smell’	14.6	0.0001	0.815	3.45	[1.02, 11.6]	0.046
Presence of ‘altered interest in sex’	9.4	0.002	0.837	2.23	[1.21, 1.42]	0.010
Presence of ‘restless legs’	10.2	0.001	0.850	3.70	[1.78, 7.63]	0.001
Presence of ‘concentration problems’	6.8	0.009	0.858	0.27	[0.12, 0.61]	0.002
NMSS score for ‘nocturia’	9.1	0.003	0.867	1.17	[1.04, 1.32]	0.007
Presence of ‘swallowing difficulty’	5.8	0.016	0.873	5.52	[1.22, 25.0]	0.027
Presence of ‘excessive sweating’	5.1	0.024	0.881^2^	3.75	[1.16, 12.1]	0.028

1 Significance level to enter  =  level to stay  = 0.05.

2 Final model AUC  = 0.881.

The NMS which discriminated between PD patients and HFS patients with statistical significance are presented in Table 6, and the symptoms that provided the greatest discriminative power were ‘constipation’, ‘restless legs’ and ‘altered interest in sex’.

**Table 6:** Selection of NMSS items by stepwise logistic regression^1^ to discriminate between PD patients (n = 200) and HFS patients (n = 75).

Selected NMSS items in the order selected	Chi-Square Score	*p*-value	Cumulative area under the ROC curve	Odds Ratio	95% Wald Confidence Intervals	Wald *p*-value
Presence of ‘constipation’	37.2	< 0.0001	0.672	37.0	[6.80, 200]	< 0.0001
NMSS score for ‘restless legs’	10.8	0.001	0.726	2.10	[1.41, 3.13]	0.001
NMSS score for ‘altered interest in sex’	10.6	0.001	0.789	1.68	[1.15, 2.46]	0.007
Presence of ‘difficulty falling or staying asleep’	8.5	0.004	0.811	0.15	[0.06, 0.36]	<0.0001
Presence of ‘weight change’	9.8	0.002	0.829	17.5	[2.45, 125]	0.004
NMSS score for ‘pain’	7.9	0.005	0.850	1.24	[1.09, 1.43]	0.002
Presence of ‘forget to do things’	6.4	0.011	0.864	0.39	[0.19, 0.80]	0.011
NMSS score for ‘dribbling saliva’	6.3	0.012	0.877	2.06	[1.24, 3.42]	0.005
Presence of ‘altered sense of taste or smell’	5.7	0.017	0.887	9.09	[1.77, 45.5]	0.008
NMSS score for ‘difficulty experiencing pleasure’	4.8	0.029	0.894	1.26	[1.05, 1.52]	0.012
Presence of ‘feel nervous’	5.2	0.023	0.897^2^	0.35	[0.14, 0.88]	0.025

1 Significance level to enter  =  level to stay  = 0.05.

2 Final model AUC  = 0.897.

Symptoms whose prevalence or burden was found to significantly discriminate between PD patients and controls as well as between PD patients and HFS patients were ‘constipation’, ‘dribbling saliva’, ‘altered sense of taste or smell’, ‘altered interest in sex’ and ‘restless legs’.

## Discussion

This is the first NMSS study to compare the NMS burden between PD patients, patients with a chronic neurological disease (hemifacial spasm, HFS), and healthy controls. We demonstrated that the overall NMS burden was significantly greater in non-demented PD patients compared to age- and sex-matched healthy controls, and increased NMS burden in PD applied to all domains of the NMSS, in line with previous studies [16], [17]. In PD patients, NMS burden was positively associated with LEDD and H&Y stage. HFS is also a chronic neurological condition, but lacks the dopaminergic or neurodegenerative pathology that causes NMS. Still, HFS can lead to sufficient distress to exert a psychological effect on patients, and anxiety symptoms have previously been shown to be increased in HFS patients [24]. Using the NMSS, we demonstrated that overall NMS burden was also increased in HFS patients compared to healthy controls. In particular, forgetting to do things, fatigue and urinary symptoms were associated with a higher burden in HFS patients compared to healthy controls, and the frequencies of sleep/fatigue and mood symptoms were also higher in HFS patients. No significant differences in the burden of ‘difficulty falling or staying asleep’ and of ‘feel nervous’ were detected between PD patients and HFS patients despite being found between PD patients and healthy controls. These could be more generic symptoms found in chronic neurological diseases that interfere with function, and therefore less specific to PD. Our results also suggest that good control of the motor symptoms in PD, the severity of which was positively associated with NMS burden, remain important in minimizing NMS. Incorporating a broader approach in empirical symptom treatment, patient education and counselling into clinical management may provide additional relief for the more generic NMS in patients with PD or other chronic neurological conditions.

On the other hand, a challenge that arises in treating generic NMS would be that of discerning the situations where the underlying cause is really intrinsic PD pathology, and early disease-specific treatment would be beneficial. The symptoms of ‘fatigue’, ‘difficulty falling or staying asleep’, ‘nocturia’ and ‘pain’ were found to contribute most to NMS burden on average in subjects regardless of disease status, and investigations and treatments for the common age-related changes or pathologies found in an elderly population may be initiated and fail in PD patients with such symptoms elsewhere before they come to the attention of their movement disorder specialists. Generic NMS resulting from intrinsic PD pathology may nevertheless have distinguishing features, an example being dystonic pain [25]. Further characterization and elucidation of features of generic NMS that better predict PD as the cause, and awareness of such features amongst physicians would aid the effective management of these NMS.

In a specialist outpatient clinic for PD patients, in addition to addressing the non-motor symptoms that are highly prevalent in PD patients and that cause the greatest distress, it is also important to address those that are most specific to PD and may be more likely to be directly caused by its pathology. Such symptoms may be either under-diagnosed or less amenable to treatment from a more accessible general practice. We used a stepwise logistic regression approach to select combinations of NMS to differentiate between i) PD patients and healthy controls, and ii) between PD patients and HFS patients. In both models, area under the ROC curve approached 0.90. In the model differentiating between PD patients and healthy controls, the burden of ‘fatigue’ had the greatest discriminative power. Fatigue may be a consequence of deficient serotonergic function in PD [26]. In PD patients, fatigue has also been associated with depression and sleep disturbances at night, which may partially explain the loss of its discriminating power for our HFS patients, who experienced more insomnia symptoms than healthy controls. Instead, the presence of ‘constipation’ (less than 3 bowel motions per week) best discriminated between PD and HFS patients, and also had the second highest contribution to the area under the ROC curve in the model discriminating between PD patients and healthy controls. In addition to ‘constipation’, symptoms of ‘restless legs’, ‘dribbling saliva’, ‘altered interest in sex’ and ‘altered sense of taste or smell’ (hyposmia) were statistically significant in both models. The NMS selected in the discriminative models that were associated with higher odds of PD could reflect a few properties: First, they may be most specific to the NMS complex directly caused by intrinsic PD pathology, and should be screened for in patients even if they are not the most prevalent or severe NMS in PD. Hyposmia and constipation, and possibly fatigue and restless legs syndrome have been implicated as symptoms in a ‘premotor’ phase of PD that may be useful symptoms for the early diagnosis of PD [27], [28]. Second, the selected items may reflect persistent NMS that are refractory to treatment, under-treated relative to the general population, or influenced by dopaminergic treatment which cannot be further optimized without compromising satisfactory control of motor symptoms. Currently, there is no available treatment for hyposmia in PD [3]. In addition, 51% of the PD patients in our study experienced constipation, despite many already being treated with laxatives. At the same time, certain NMS may have tended not to be selected in the discriminant models: The NMSS only rates symptoms that occurred in the last month, and may not capture symptoms that occur only transiently or intermittently (at intervals of more than one month) in PD patients. For example, symptoms of hallucinations, orthostatic hypotension and sleep disturbances may be relieved by the adjustment of PD medications [29]. Certain NMS selected in the discriminative model were associated with reduced odds for PD, and it is possible that these were artifacts in our dataset – their contribution to area under the ROC curve was small.

This study had limitations. First, the NMSS scale is not intended for diagnosis and relies on the subjective perception of symptom severity by subjects [14]. However, it is ideal for assessing a large number of symptoms in a reasonable amount of time while minimizing responder fatigue. Second, PD patients with significant cognitive impairment were excluded, preventing us from making conclusions about this subpopulation of patients. Certain NMS, such as apathy, hallucinations and depression have been associated with cognitive impairment and may have not featured strongly in this study [1], [30]–[33]. Third, as the disability faced by patients in the HFS group is less severe than that experienced by PD patients, we were unable to attribute the higher NMS burden in PD patients compared to HFS patients entirely to intrinsic PD pathology in this study. In addition, it would be useful to obtain quality-of-life (QoL) data for both PD and HFS patients to explore how NMS may have a different impact on QoL in PD as opposed to HFS. Last, spouses of PD patients also participated as healthy controls, and we may have underestimated the increased NMS burden in PD patients if these controls’ sleep, fatigue levels and/or mood were influenced by their spouses who had PD.

In conclusion, our study demonstrated a greater burden of non-motor symptoms in non-demented PD patients compared to healthy controls. We showed that certain NMS may be common to chronic neurological diseases and lack specificity for PD, whereas the symptoms of ‘constipation’, ‘restless legs’, ‘dribbling saliva’, ‘altered interest in sex’ and ‘altered sense of taste or smell’ discriminated PD patients from both healthy controls and HFS patients. Clinicians should have a high index of suspicion for NMS in PD patients, and awareness of which symptoms are common to chronic neurological disorders and which are more specific to PD may help in refining treatment strategies. These would enhance the clinical management of PD patients.
